# Supplementary material for: Large language model reveals an increase in climate contrarian speech in the United States Congress
Source: Commun Sustain. 2026 Feb 27;1(1):37. doi: 10.1038/s44458-025-00029-z (PMC12983461; doi:10.1038/s44458-025-00029-z)
Supplement: Supplementary file 2 — Supplementary Information [file 44458_2025_29_MOESM2_ESM.pdf]

Supplementary Information

S.1 Supplementary Methods: Taxonomy revision

In revising the CARDS taxonomy, we followed a two-phase approach: the first phase focused on taxonomy comprehensiveness and logical coherence, the second on optimizing the hierarchy for machine classification.

Phase 1: Enhancing Taxonomy Comprehensiveness and Logical Coherence

We began by labeling contrarian witness testimonies at congressional hearings on climate change legislation. This exercise allowed us to iteratively extend and reorganize the existing hierarchy through the following methods:

- **Translating delay strategies into content claims.** We reformulated tactics identified in the Discourses of Climate Delay framework as discrete content claims, mapping them onto existing claims within the taxonomy or introducing new claims where necessary (see Tab. 1).
- **Incorporating previously undocumented claims.** We included additional recurring policy-related arguments encountered during labeling—absent from both original frameworks—as new level-2 claims.
- **Removing obsolete claims.** We dropped the claims *Carbon Capture and Sequestration is unproven* and *Nuclear is good*, as these are no longer treated as distinct claims within the taxonomy.
- **Refining hierarchical coherence.** We adjusted parent–child relations to ensure each category had a clearly delimited scope, thereby reducing overlap and ambiguity.

Super-claims 1, 2, 3, and 5 required minimal adjustments, as they already provided broad and coherent coverage. In contrast, we substantially reorganized the policy-centered super-claim 4 to accommodate the expanded array of policy-related arguments.

Phase 2: Optimizing the Hierarchy for Machine Classification

During classifier training, we identified further shortcomings in claim discrimination, prompting additional refinements to the hierarchy:

- **Tightening claim definitions.** We formulated and sharpened definitions for each claim—particularly where conceptual overlap persisted—to ensure sibling categories were mutually exclusive and more readily distinguishable by the model.
- **Removing redundancy.** Where overlap between level-2 claims persisted, we consolidated separate level-2 claims into one.
- **Splitting level-1 category 5.** To separate critiques of scientific evidence and methodology from ad hominem or institutional attacks, we divided the original super-claim 5 into two distinct level-1 claims:
  - **Level-1 claim 5:** Climate-related science is uncertain
  - **Level-1 claim 6:** Proponents of climate action are alarmists
- **Elevating fossil fuel advocacy to a new major claim.** We promoted the argument that people need energy from a level-2 claim under policy-related arguments to form its own level-1 claim. While this argument is often employed to oppose climate policy, it also represents a broader normative stance on energy requirements, thus justifying its separation.
  - **Level-1 claim 7:** We need fossil fuels

These adjustments substantially improved the classifier's ability to assign accurate labels across the revised taxonomy, resulting in a more robust and discriminative classification system.

Table S1. Updated CARDS taxonomy

Claim	Short label	Prompt label
1_0_0	Global warming is not happening	Global warming is not happening. Climate change is NOT leading to melting ice (such as glaciers, sea ice, and permafrost), increased extreme weather, or rising sea levels. Cold weather also shows that climate change is not happening.
1_1_0	Ice/permafrost/snow cover isn't melting	Climate change is NOT causing melting ice (e.g., glaciers or sea ice), thawing permafrost, or reduced snow cover.
1_1_1	Antarctica is gaining ice/not warming	Antarctica is gaining ice.
1_1_2	Greenland is gaining ice/not melting	Greenland is gaining ice.
1_1_3	Arctic sea ice isn't vanishing	Arctic sea ice is not vanishing.
1_1_4	Glaciers aren't vanishing	Glaciers are not melting and may be gaining mass.
1_2_0	We're heading into an ice age/global cooling	We are heading into a period of global cooling or an ice age.
1_3_0	Weather is cold/snowing	We are experiencing cold weather, therefore climate change is not happening.
1_4_0	Climate hasn't warmed/changed over the last (few) decade(s)	There has been a hiatus or pause in global warming. The climate has not warmed or changed very much over the past few decades.
1_5_0	Oceans are cooling/not warming	Oceans are not warming and may even be cooling.
1_6_0	Sea level rise is exaggerated/not accelerating	Sea level rise is exaggerated and not accelerating.
1_7_0	Extreme weather isn't increasing/has happened before/isn't linked to climate change	Climate change does not cause or worsen extreme weather events such as heatwaves, droughts, wildfires and floods.
1_8_0	They changed the name from 'global warming' to 'climate change'	Climate advocates and alarmist changed the name from global warming to climate change so that cold weather as well as hot can be taken as evidence.
1_9_0	Ocean pH is not falling	Oceans are not becoming more acidic and ocean pH is not falling.
2_0_0	Human greenhouse gases are not causing climate change	Greenhouse gases from humans are not the causing climate change.
2_1_0	It's natural cycles/variation	Humans are not causing change. Instead, climate change is due to natural variation.
2_1_1	It's the sun/cosmic rays/astronomical	The sun, cosmic rays, or other astronomical phenomena are causing climate change.
2_1_2	It's geological (includes volcanoes)	Geological events such as volcanic activity are causing climate change.
2_1_3	It's the ocean/internal variability	Natural ocean variability is causing climate change, not manmade factors. This natural variability includes El Nino and La Nina climate patterns.
2_1_4	Climate has changed naturally/been warm in the past	Climate has changed naturally and/or it's been warm in the past, so we shouldn't worry too much about recent climate change.
2_2_0	It's non-greenhouse gas human climate forcings (aerosols, land use)	Non-greenhouse gas human climate forcings such as from aerosols, changes in land use, or black soot on snow are causing climate change.
2_3_0	There's no evidence for greenhouse effect/carbon dioxide driving climate change	There's no evidence for greenhouse effect or carbon dioxide driving climate change
2_3_1	Carbon dioxide is just a trace gas	CO_2 is just a trace gas and so can't cause climate change.
2_3_2	Greenhouse effect is saturated/logarithmic	The greenhouse effect is saturated (or logarithmic) and therefore will have little effect on climate.
2_3_3	Carbon dioxide lags/not correlated with climate change	Temperature changes drive carbon dioxide, not the other way around.

Note: Numeric labels of new claim additions highlighted in bold.

Table S1. Updated CARDS taxonomy (continued)

Claim	Short label	Prompt label
2_3_4	Water vapor is the most powerful greenhouse gas	Naturally occurring water vapor is the most powerful greenhouse gas.
2_3_5	There's no tropospheric hot spot	There's no tropospheric hot spot.
2_3_6	CO_2 is not rising.	CO_2 is not rising.
2_3_6_1	CO_2 was higher in the past	CO_2 concentrations were higher in the past
2_3_6_2	Human CO_2 emissions are minuscule/not raising atmospheric CO_2	CO_2 emissions from humans are tiny and/or not raising atmospheric CO_2
3_0_0	Climate impacts/global warming is beneficial/not bad	The impacts of climate change will not be bad and might even be beneficial.
3_1_0	Climate sensitivity is low/negative feedbacks reduce warming	Climate sensitivity is low and there are negative feedbacks that will reduce warming.
3_2_0	Species/plants/reefs aren't showing climate impacts yet/are benefiting from climate change	Plants and animals are not showing harmful impacts from climate change and may be benefiting from climate change
3_2_1	Species can adapt to global warming	Plants and animals will adapt to climate change and therefore the impacts will be minimal.
3_2_2	Polar bears are not in danger from climate change	Polar bears are not in danger from climate change.
3_2_3	Ocean acidification/coral impacts aren't serious	The impact of ocean acidification on coral is exaggerated. The oceans are and have been alkaline in the past, yet coral has survived.
3_3_0	CO_2 is beneficial/not a pollutant	CO_2 is not a pollutant.
3_3_1	CO_2 is plant food	CO_2 is plant food – it helps plant growth.
3_4_0	It's only a few degrees (or less)	Human-caused climate change will only lead to a few degrees (or less) of warming and so no cause for alarm.
3_5_0	Climate change does not contribute to human conflict/threaten national security	Climate change does not lead to conflict and is not a national security threat.
3_6_0	Climate change doesn't negatively impact health	Climate change does not have negative impacts on human health.
4_0_0	Climate solutions won't work	Climate solutions are harmful or unnecessary
4_1_0	Climate solutions are harmful	Climate solutions are harmful to the economy, society, and/or the environment
4_1_1	Solutions increases costs	Climate solutions will increase costs, harm the economy, and/or kill jobs
**4_1_1_1**	Policy harms competitiveness	Climate policies will harm economic competitiveness
**4_1_1_2**	Policy harms vulnerable members of society	Climate policies will kill jobs and/or harm vulnerable members of society
**4_1_1_3**	Climate-friendly alternatives are too expensive	Climate-friendly technologies and practices are too expensive and/or uneconomical
4_1_2	Policy weakens security	Climate policies will weaken national security, energy security, national sovereignty, and/or cause conflict
4_1_3	Solutions harm environment	Climate solutions will harm the environment, habitats, and/or species
**4_1_3_1**	Policy harms environment	Government climate policies will harm the environment, habitats, and/or species
**4_1_3_2**	Climate-friendly alternatives harm environment	Climate-friendly technologies/practices will harm the environment, habitats, and/or species
**4_1_4**	Policy creates uncertainty	Climate policies create economic uncertainty and may have unintended consequences

Note: Numeric labels of new claim additions highlighted in bold.

Table S1. Updated CARDS taxonomy (continued)

Claim	Short label	Prompt label
4_1_5	Policy limits freedom	Climate regulation limits individual liberty, freedom, and undermines capitalism. This includes but not limited to arguments that climate solutions are a justification for government overreach and control. Note that claims of a "war on energy" would fall into this category.
4_2_0	Climate solutions are ineffective	Climate solutions are ineffective and won't work.
4_2_1	Green economy won't work	The promised benefits of green jobs won't be achieved in practice.
4_2_2	Policy impact is negligible	The impact of climate policies on climate change will be negligible and/or only make a difference in the distant future
4_2_3	One country is negligible	A single country or region only contributes a small percentage of global emissions
4_2_4	Other countries' emissions	Climate action is pointless because of the emissions of other countries such as China or India
**4_2_6**	Policies can be manipulated	Climate policy can be gamed or manipulated.
4_2_7	Climate-friendly alternatives are ineffective	Climate-friendly technologies and practices are ineffective and won't work.
**4_2_7_1**	Not ready	Climate-friendly technologies are not ready
**4_2_7_2**	Not enough	Renewable energy cannot provide base-load power and is difficult to scale
4_2_8	Markets are more efficient	Markets and private sector are economically more efficient than government policies at solving climate change
**4_2_9**	Individuals are responsible	Individuals/consumers are responsible for climate change and should change their behavior to solve it
4_2_10	Future generations, technologies, and efficiencies will solve it	Future generations, technologies, and efficiencies will solve it
**4_2_10_1**	Future generations will fix it	Future generations will be richer and will be better able to solve climate change
**4_2_10_2**	Technology will fix it	Future technology will fix climate change, so we shouldn't worry too much.
4_2_11	Adaptation is the solution	It's better to adapt to climate change and increase resiliency then to devote resources to mitigation.
**4_2_12**	Energy efficiency is enough	Increasing energy efficiency is enough to meet the challenges of climate change.
**4_2_13**	Removing CO_2 is the solution	We should focus on carbon dioxide removal rather than emissions reductions
**4_2_14**	Other issues are more pressing	There are more pressing problems than climate change and we should address those first
**4_2_15**	Cheaper to mitigate abroad	It's cheaper to mitigate climate change abroad, so we are better off focusing on helping other countries reduce their emissions
4_3_0	Solving climate change is too difficult	It's too hard to solve climate change and so we shouldn't try
**4_3_1**	We're not ready for policy	We don't have the right policy to solve climate change and/or need to better understand all the implications before implementing climate policies
**4_3_2**	It's too late to fix it	It's too late to do anything to mitigate climate change
4_3_3	Low support	Support for climate solutions is low and/or decreasing
**4_4_0**	No need for more action	We have already made a lot of progress on climate change and don't need to do anything else.
**4_4_1**	Already taking it seriously	We are already taking climate change seriously, so there is no need to worry
**4_4_2**	Already doing enough	We are already doing enough to address climate change, so there is no need for more action

Note: Numeric labels of new claim additions highlighted in bold.

Table S1. Updated CARDS taxonomy (continued)

Claim	Short label	Prompt label
**4_4_3**	Already doing good	We are already contributing enough to the societal good we don't need to also address climate change issues
5_0_0	Climate-related science is uncertain/unsound/unreliable (data, methods & models)	Climate science is uncertain, unsound, unreliable, or biased.
5_1_0	There's no scientific consensus on climate/the science isn't settled	There is no scientific consensus on climate change. Scientists continue to disagree on many aspects of climate change and the science is not settled. This includes arguments that the science isn't settled or isn't there.
5_2_0	Proxy data is unreliable (includes hockey stick)	Proxy climate data from things such as ice cores, tree rings boreholes, etc., are unreliable. This includes "hockey stick" graph.
5_3_0	Temperature record is unreliable	Temperature data is unreliable and/or biased.
5_4_0	Models are wrong/unreliable/uncertain	Climate models are flawed, unreliable, or uncertain.
6_0_0	Proponents are alarmist	Climate scientists and proponents of climate action are alarmist, biased, wrong, hypocritical, corrupt, and/or politically motivated.
6_1_0	Climate movement is alarmist/wrong/political/biased/hypocritical (people or groups)	Climate change proponents are alarmist, biased, wrong, hypocritical, and/or politically motivated.
6_1_1	Climate movement is a religion	Climate movement is a religion. Belief in climate change is based on faith, and not scientific evidence
6_1_2	Media (includes bloggers) is alarmist/wrong/political/biased	Media reports on climate change are alarmist, biased, and/or wrong.
6_1_3	Politicians/government/UN are alarmist/wrong/political/biased	Politicians, governments, and organizations such as the UN are alarmist, biased, and/or wrong on climate change.
6_1_4	Environmentalists are alarmist/wrong/political/biased	Environmentalists are alarmist, biased, and/or wrong on climate change.
6_1_5	Scientists/academics are alarmist/wrong/political/biased	Scientists and academics are alarmist, biased, and/or wrong on climate change.
6_2_0	Climate change (science or policy) is a conspiracy (deception)	Climate change is a hoax or conspiracy. We have been deceived by climate scientists, politicians, bureaucrats, and environmental organizations on climate change.
7_0_0	We need fossil fuels	We need fossil fuels for economic growth, prosperity, and to maintain our standard of living.
7_1_0	Fossil fuels are plentiful	Fossil fuels are plentiful and should be used. This includes arguments that explicitly focus on large domestic fossil fuel reserves or an abundance of potential fossil fuel resources.
7_2_0	Fossil fuels are good	Fossil fuels are good for the economy, society, and/or the environment.
**7_2_1**	Good for economic growth	Fossil fuels are important for economic growth and development. Only assign when text explicitly links fossil fuels to economic growth/development.
**7_2_2**	Good for energy security	Fossil fuel extraction is important for energy security. Assign when text emphasizes the importance of fossil fuels for domestic security or energy independence.
**7_2_3**	Our fossil fuels are clean	Our fossil fuels are cleaner than others
**7_2_4**	Fossil fuels are part of the solution	Fossil fuels are part of the solution. We need to transition to cleaner fossil fuels.
**7_3_0**	Fossil fuels are necessary	Fossil fuels are necessary to meet energy demand. This includes, but not limited to, arguments that we need all forms of energy, including fossil fuels.

Note: Numeric labels of new claim additions highlighted in bold.

Table S1. Updated CARDS taxonomy (continued)

Claim	Short label	Prompt label
**7_4_0**	We have the right to use them	We have a right to profit from fossil fuels just like others have
0_0_0	No Claim detected	No relevant claim detected

Note: Numeric labels of new claim additions highlighted in bold.

S.2 Supplementary Methods: Robustness of empirical results to speech length

Table S2. Regression Results: Full Model and Republican Model

	Full Model		Republican Model	
Variable	Estimate	95% CI	Estimate	95% CI
Intercept	−4.92	[−5.347, −4.498]	−3.846	[−4.349, −3.342]
Republican	2.634	[2.461, 2.812]	—	—
Independent	−0.781	[−2.77, 0.907]	—	—
Senate	−0.287	[−0.458, −0.119]	−0.332	[−0.505, −0.155]
Female	−0.565	[−0.814, −0.328]	0.011	[−0.26, 0.278]
Age	−0.161	[−0.23, −0.091]	−0.11	[−0.182, −0.04]
FF Contributions	0.01	[−0.018, 0.037]	0.007	[−0.022, 0.036]
FF Employment	0.237	[0.174, 0.3]	0.138	[0.076, 0.2]
Democrat in Power	0.469	[−0.033, 0.963]	0.492	[−0.055, 1.036]
Total Words Spoken	−0.077	[−0.144, −0.011]	−0.07	[−0.143, 0.004]
Ideology	—	—	1.485	[1.264, 1.707]

S.3 Supplementary Methods: Fossil fuel employment data

The creation of constituency-level **fossil fuel employment** variables involved a multi-stage data processing pipeline that combined Bureau of Labor Statistics (BLS) employment data with congressional district shapefiles across multiple congressional terms. We obtained county-level employment data from the BLS' Quarterly Census of Employment and Wages (QCEW) database, which covered the period 1990-2023. We first employed a Python script to automate the download of annual industry data files from the BLS. We then filtered the comprehensive industry dataset to focus on specific NAICS codes associated with fossil fuel extraction and related activities: oil and gas extraction (211), coal mining (2121), drilling oil and gas wells (213111), support activities for oil and gas operations (213112), support activities for coal mining (213113), fossil fuel electric power generation (221112), natural gas distribution (2212), and pipeline transportation of crude oil, natural gas, refined petroleum products and other products or slurry, including the storage of natural gas (486).

A significant challenge involved handling the heterogeneous formats of congressional district shapefiles across different time periods. Congressional district boundaries change every decade following redistricting (and in some states more than once a decade). Our approach addressed these complexities through a systematic shapefile management system. For each congressional term (103rd-118th Congress), the system automatically identified the appropriate shapefile: for the 103rd-114th Congress, we used historical district shapefiles from Jeff Lewis et al's redistricting database[4], with a few alterations in some shapefiles to correct known geometry issues; for the 115th-116th Congress, we used Census Tiger/Line shapefiles; for the 117th Congress, we used ESRI's Congressional Districts geodatabase; and for the 118th Congress, we used Census Cartographic Boundary files. All shapefiles were reprojected to EPSG:5070 (Albers Equal Area Conic) to ensure accurate area calculations during spatial operations.

The core spatial analysis involved overlaying county-level employment data with congressional district boundaries using geometric intersection methods. For each congressional term, county polygons containing fossil fuel employment data were intersected with district boundaries to determine which portions of each county fell within each district. When counties crossed district boundaries, employment statistics were allocated proportionally based on the area of intersection. For example, if 30% of a county's area fell within a particular district, then 30% of that county's fossil fuel employment was attributed to that district. County-level statistics allocated to each district were then summed to create district-total measures of fossil fuel employment, which were then normalized by district total employment.

S.4 Supplementary Methods: ClimateBERT

ClimateBERT is a family of language models created by fine-tuning the DistilRoBERTa model on a dataset comprising climate-related corpora, general news articles, and corporate reports[1]. We employed the fine-tuned ClimateBERT model with a classification head to detect climate-related paragraphs in our congressional dataset. Given the dataset's size (over 2.5 million paragraphs), this filtering step was essential to reduce computational costs associated with subsequent large language model API calls. ClimateBERT identified 110,837 paragraphs as climate-related, which we then processed using our CARDS framework.

S.5 Supplementary Methods: Evolution of our Prompts

Prompt engineering is an iterative, bi-directional exercise, and the best way to combine human and machine intelligence in the context of GenAI. Some of the best practices according to Anthropic's and OpenAI's documentation in developing a "good" prompt are (https://docs.anthropic.com/en/docs/build-with-claude/prompt-engineering/overview)

1. Assigning a role to the system and having a clear objective/task definition
2. Use XML tags for clarity, accuracy, flexibility (to add or remove new content), and the ease with which they allow one to parse responses.
3. Place the long context before giving the instructions.
4. Let the models "Think" - Chain of thought prompting and omit the steps in order to reach the final response.

Chain-of-Thought: According to https://arxiv.org/abs/2211.01910, the Automatic prompt engineer outperformed all other chain of thought triggers. So, we proceeded with the following trigger for this analysis
Let's work this out in a step by step way to be sure we have the right answer.

By taking all the practices into account, our initial **system prompt** is rather simple and straightforward to begin with.

System Instruction (system prompt)

Version 1

You are an expert on climate communication. Your task is to classify the given text into categories based on the codebook provided to you. This is a multi-label classification.

TEXT:
{text}

CODEBOOK:
{codebook}

IMPORTANT:

```
1. If you think the text does not belong to any of the categories in the codebook,
   and the text is not expressing any skepticism towards climate change or
   climate action, please use the following category:
   <0_0_0>No relevant claim detected.<0_0_0/>
2. Only focus on parts of the text that are associated with climate change or
   climate action. Ignore any other parts of the text.
3. Strictly adhere to the desired output format. Any deviation from the desired
   output format will result in a failed evaluation.
4. Don't just look for relevance, but check if the text agrees with the category
   in the codebook. If the text does not agree with the category, do not assign
   the category to the text.
```

DESIRED OUTPUT FORMAT:

```
{
  "categories": [
    {
      "category": "<category_code, a XML tag>",
      "text": "<text_associated_with_the_category>"
    },
    {
      "category": "<category_code, a XML tag>",
      "text": "<text_associated_with_the_category>"
    }
    ...
  ]
}
```

As we mentioned that prompt engineering is a bi-directional and iterative process, we have created a ChatBot to test the effectiveness of the prompt in each iteration. Based on the interactions from the ChatBot, it was clear that the model's understanding of the task is quite good, but the context is way too limited for it to make an informed decision. Thanks to the Chain of thought prompting, we can actually read the step by step reasoning of the model before it arrives at a decision. A team of coders interacted with the Bot and noted down their feedback on how and why the model is making an incorrect classification.

Version 2

You are an expert on climate communication. Your task is to classify the given text into categories based on the codebook provided to you. This is a multi-label classification.

TEXT:
{text}

CODEBOOK:
{codebook}

IMPORTANT INSTRUCTIONS:

```
1. **Thoroughly Review All Categories**:
   - Carefully check the text against all categories in the codebook to ensure
     comprehensive classification.

2. **Understand the Codebook Structure**:
   - The codebook is a hierarchical structure.
   - Each category has a unique XML tag associated with it.
   - Superclaims' XML tags end with `_0_0`, subclaims end with `_0`.

3. **Hierarchical Classification Process**:
   - **Step 1**: Compare the text against all superclaims to identify the most relevant
     ones.
   - **Step 2**: For each relevant superclaim, compare the text against its subclaims to
     identify the most relevant subclaims.
   - **Step 3**: Continue this process until you reach the most granular level of the
     codebook.
   - **Note**: Always return the most granular level that the text belongs to.

4. **Focus on Precision and Recall**:
   - Your primary goal is to respond with high precision and recall.
   - Do not leave any relevant claim unassigned.
   - Do not assign any irrelevant claim to the text.

5. **Accuracy is Crucial**:
   - An expert in the field will review your response.
   - Ensure your response is as accurate as possible.

6. **Handling Irrelevant Text**:
   - If the text does **NOT** belong to any of the categories and does **NOT** express
     skepticism towards climate change, climate solutions, promotes fossil fuels, or attacks
     renewable energy, then use the category `<0_0_0>No relevant claim detected.</0_0_0>`.
   - Category `<0_0_0>No relevant claim detected.</0_0_0>` is mutually exclusive with all
     other categories.
   - If the text looks incomplete, unclear, or no sufficient information is provided, use
     the category `<0_0_0>No relevant claim detected.</0_0_0>`.

7. **Focus on Relevant Content Only**:
   - Concentrate solely on parts of the text associated with climate change, climate
     solutions, energy (i.e., fossil fuels or renewable energy).
   - Ignore any parts of the text that are not related to these topics.
```

```

8. **Special Attention to Fossil Fuels**:
   - If the text suggests the necessity or benefits of fossil fuels, carefully check
     against all claims starts with 7_.

9. **Strictly Adhere to Output Format**:
   - Any deviation from the desired output format will result in a failed evaluation.
   - Do not include any additional information outside the specified format.

10. **Ensure Agreement with Categories**:
    - Assign a category only if the text agrees with it.
    - Do not assign a category if the text does not support it.

11. **Review**:
    - If your confidence in the classification is low, you can mark the review field as
     'yes' to indicate that your response requires further review. But you should still
     provide your best classification.
```

DESIRED OUTPUT FORMAT:

```
{
  "review": "<Yes/No>",
  "categories": [
    {
      "category": "<category_code, a XML tag>",
      "text": "<text_associated_with_the_category>"
    },
    {
      "category": "<category_code, a XML tag>",
      "text": "<text_associated_with_the_category>"
    }
    ...
  ]
}
```

The above system prompt has a clear, extensive set of instructions, emphasis on the codebook being a hierarchical one, and provides extra information on how to handle irrelevant text (this includes non-claims), any text related to fossil fuels (quite frankly, this is one of the harder ones to classify). Finally, we added a new key in the output format json, and asked the model to ask for a review in case it is not very confident with its decision. The idea is in an Agentic universe, when our finetuned-model asks for a review, it will be sent to a top frontier model (such as o1, sonnet-3.5, GPT-4o, deepseek-v3, etc).

Evaluating and optimizing prompts for language models requires significant collaborative effort between programmers and domain experts. To streamline this process, we developed an interactive chatbot interface that implements our classification system's prompts and codebook. This solution allows multiple users to simultaneously test and evaluate the system without requiring direct programmer involvement.

The chatbot interface was tested using our prompt version minus 1, which included hierarchical classification guidelines and specific handling instructions for irrelevant text and fossil fuel-related content. The system was also designed to request expert review when confidence levels fell below a certain threshold, leveraging more advanced language models (such as Claude 3.5 Sonnet, GPT-4, or DeepSeek v3) for verification. Initial testing revealed several key challenges, as outlined in Table S3.

Category	Limitation	Mitigation Strategies
Incomplete Classifications	The model frequently failed to identify all applicable claims within a given text, resulting in partial responses.	Enhanced the system by implementing comprehensive cross-referencing mechanisms and providing targeted hints for complex classification categories.
Insufficient Granularity	Despite instructions to prioritize lowest-level classifications within the hierarchy, the model showed inconsistent adherence to this requirement.	Implemented explicit hierarchical navigation guidelines, including decision trees and specific criteria for moving between hierarchical levels.
Classification Ambiguity	The model struggled to differentiate between closely related claims and to distinguish between mere reporting of information versus endorsement of claims.	Developed comprehensive disambiguation guidelines with explicit criteria for identifying implicit endorsements and contextual markers.
Accuracy Issues	Both false positives and false negatives were observed in the classification results.	Implementation of the above solutions is expected to reduce classification errors through more precise criteria and improved context understanding.

Table S3. Model Limitations and Solutions

By incorporating all the mitigation strategies into our system prompt, it has evolved into the version below.

S.5.1 Version 3

You are an expert on climate communication. Your task is to classify the given text into categories based on the codebook provided to you. This is a multi-label classification.

CODEBOOK:
{codebook}

IMPORTANT INSTRUCTIONS:

```
1. **Thoroughly Review All Categories**:
   - Carefully check the text against all categories in the codebook to ensure
    comprehensive classification.

2. **Understand the Codebook Structure**:
   - The codebook is a hierarchical structure.
   - Each category has a unique XML tag associated with it.
   - Superclaims' XML tags end with `_0_0`, subclaims end with `_0`.

3. **Hierarchical Classification Process**:
   - **Step 0**: First check if the text fits into the category `<0_0_0>No relevant claim
    detected.</0_0_0>`. If it does, assign this category and ignore all other categories.
    This category is mutually exclusive with all other categories.
   - **Step 1**: Compare the text against all superclaims to identify the most relevant
    ones.
   - **Step 2**: For each relevant superclaim, compare the text against its subclaims to
    identify the most relevant subclaims.
   - **Step 3**: Continue this process until you reach the most granular level of the
    codebook.
   - **Note**: Always return the most granular level that the text belongs to.

4. **Focus on Precision and Recall**:
   - Your primary goal is to respond with high precision and recall.
   - Do not leave any relevant claim unassigned.
   - Do not assign any irrelevant claim to the text.
```

5. **Accuracy is Crucial**:
   - An expert in the field will review your response.
   - Ensure your response is as accurate as possible.

6. **Handling Irrelevant Text**:
   - If the text does **NOT** belong to any of the categories and does **NOT** express skepticism towards climate change, climate solutions, promote fossil fuels, or attacks renewable energy, then use the category ‘<0_0_0>No relevant claim detected.</0_0_0>‘.
   - Category ‘<0_0_0>No relevant claim detected.</0_0_0>‘ is mutually exclusive with all other categories.
   - If the text looks incomplete, unclear, or no sufficient information is provided, use the category ‘<0_0_0>No relevant claim detected.</0_0_0>‘.

7. **Focus on Relevant Content Only**:
   - Concentrate solely on parts of the text associated with climate change, climate solutions, energy (i.e., fossil fuels or renewable energy).
   - Ignore any parts of the text that are not related to these topics.

8. **Special Attention to Fossil Fuels**:
   - If the text suggests the necessity or benefits of fossil fuels, carefully check against all claims beginning with 7_.

9. **Grouping of Superclaims for Quick Reference**:

    1. **Temperature & Weather Patterns**:
    - **Codes**: ‘1_0_0‘ to ‘1_8_0‘
    - **Focus**: Addresses skepticism around warming trends, cooling predictions, pauses in warming, and specific climate events like cold weather, sea level rise, extreme weather, and name changes from ‘global warming‘ to ‘climate change.‘

    2. **Human vs. Natural Causes**:
    - **Codes**: ‘2_0_0‘ to ‘2_3_6‘
    - **Focus**: Discusses the cause of climate change, contrasting human-induced (e.g., greenhouse gases) vs. natural factors (e.g., solar activity, volcanic activity, natural ocean variability, historical climate changes). This also covers arguments denying the greenhouse effect and minimizing the role of CO$_{2}$.

    3. **Impacts of Climate Change**:
    - **Codes**: ‘3_0_0‘ to ‘3_6_0‘
    - **Focus**: Claims about the impacts of climate change, including potential benefits, low climate sensitivity, and adaptation by plants, animals, and humans. Covers specific impacts (or lack thereof) on human health, ecosystems, and security.

    4. **Economic & Societal Impacts of Solutions**:
    - **Codes**: ‘4_0_0‘ to ‘4_4_3‘
    - **Focus**: Examines claims that climate solutions are either harmful or unnecessary, with emphasis on economic costs, environmental harms, personal freedoms, and doubts about solution effectiveness. This includes economic competition, job impacts, national security, and arguments about adaptation vs. mitigation.

    5. **Climate Science Reliability**:
    - **Codes**: ‘5_0_0‘ to ‘5_4_0‘
    - **Focus**: Questions the reliability of climate science, including arguments about scientific consensus, proxy data reliability (like ice cores and tree rings), temperature data biases, and the accuracy of climate models.

    6. **Critique of Climate Action Proponents**:
    - **Codes**: ‘6_0_0‘ to ‘6_2_0‘
    - **Focus**: Criticizes climate action advocates, portraying them as alarmist, biased,

```
corrupt, or politically motivated. This section also includes claims about climate
change as a `religion,` biased media reporting, and political manipulation.

 7. **Arguments Around Fossil Fuels**:
 - **Codes**: `7_0_0` to `7_4_0`
 - **Focus**: Highlights arguments supporting fossil fuel use, emphasizing economic
growth, energy security, environmental benefits, and the necessity of fossil fuels for
meeting energy demand.

 8. **No Relevant Claim Detected**:
 - **Code**: `0_0_0`
 - **Focus**: Use this only if the text does not contain any claims relevant to climate
change skepticism, climate solutions, fossil fuel advocacy, or renewable energy
critiques.

10. **Cross-Reference Hints for Common Themes**:
   - Pay special attention to overlapping themes, particularly between economic impacts
   (4_X_X) and fossil fuel benefits (7_X_X) AND science is uncertain (5_X_X) and
   proponents are alarmist or corrupt (6_X_X).
  - If the text mentions **global cooling or natural climate variations**, also consider
   claims about **natural drivers** like **solar influence, ocean cycles, and past climate
   change** (e.g., 2_1_0, 2_1_1, 2_1_3).
   - If the text refers to **economic impacts of climate solutions**, check both **4_1_0**
   for direct economic concerns and **7_0_0 series** if it emphasizes the need for fossil
   fuels in supporting the economy.
   - Arguments about **renewable energy feasibility** may overlap with **4_2_7_2**
   (renewables' base-load challenges) and **7_3_0** (fossil fuels for energy security).

11. **Granularity Rule**:
    - When a text matches both a parent category and its subcategories, ONLY include the
    most specific subcategories
    - Example: If text matches both 4_1_1 and 4_1_1_1, only include 4_1_1_1

12. **Distinguish Between Description and Endorsement**:
    - Carefully differentiate between texts that DESCRIBE skeptical arguments and texts
    that MAKE skeptical arguments
    - Only classify claims that the text actively endorses or promotes
    - Meta-commentary or criticism of skeptical arguments should be classified as <0_0_0>

13. **Strictly Adhere to Output Format**:
   - Any deviation from the desired output format will result in a failed evaluation.
   - Do not include any additional information outside the specified format.
   - Don't try to fit the text into a category if it doesn't align with the claim. In other
   words, don't force or stretch the text to fit a category.
```

DESIRED OUTPUT FORMAT:

```
{
    "review": "<yes/no>",
    "categories": [
      {{
        "category": "<category_code, an XML tag>",
        "text": "<text_associated_with_the_category>"
      }},
      {{
        "category": "<category_code, an XML tag>",
        "text": "<text_associated_with_the_category>"
      }}
      // ... additional categories as needed
    ]
  }
```

This version of the prompt addressed all the limitations we have recorded and yielded much better results during our prompt testing than all previous versions. So, we proceeded to run our experiments with this version of the system prompts.

S.6 Supplementary Methods: Reverse engineered chain-of-thought prompting

Knowledge distillation is a machine learning technique where a smaller "student" model learns to mimic the behavior of a larger "teacher" model. It was originally developed for compressing neural networks[3], and has been adapted for large language models to transfer reasoning capabilities from teacher to student models. Recent approaches focus specifically on chain-of-thought reasoning: Symbolic Chain-of-Thought Distillation (SCoTD) trains smaller models on multiple reasoning chains sampled from large teachers[5], while implicit approaches distill explicit reasoning into internal hidden states[2]. However, these methods share a fundamental limitation: teachers generate reasoning chains while already knowing the correct answers, which can lead to post-hoc rationalizations rather than authentic analytical processes. This issue is particularly problematic for complex classification tasks where reliable step-by-step reasoning is essential.

We developed Reverse Engineered Chain-of-Thought (RECoT) prompting to address this limitation while leveraging expert domain knowledge. RECoT instructs state-of-the-art teacher models (Claude 3.5 Sonnet and GPT-4o) to analyze labeled examples and generate step-by-step reasoning that leads to researcher-provided classifications. The key innovation is a simple but important constraint: the models must approach each example as if they have not been given the correct answers. This approach captures genuine analytical thinking rather than explanations constructed with knowledge of the solution.

The RECoT procedure works in three stages. First, we compile a high-quality dataset with expert-annotated examples using our AI-assisted annotation tool. Second, we prompt teacher models with a specialized system prompt to generate reasoning chains for each example, explicitly instructing them to reason through the problem independently before arriving at the provided answer. Finally, we fine-tune the target model (GPT-4o-Mini) on the complete reasoning chains generated by the teachers, enabling it to learn both the reasoning process and the down-stream classification task.

RECoT's focus on reasoning authenticity delivers substantial performance improvements. Our experiments demonstrate that RECoT-trained GPT-4o-Mini (i.e., CARDS-mini-Sonnet-2024-12-05) achieves a 27.5% improvement in F1 score compared to the base model, while producing more interpretable and reliable reasoning chains. Unlike Symbolic Chain-of-Thought Distillation, which samples multiple forward reasoning chains from teachers[5], or implicit approaches that internalize reasoning within hidden states[2], RECoT specifically targets the quality of reasoning generation. The method transforms smaller models that typically struggle with consistent chain-of-thought reasoning, demonstrating that authentic reasoning generation can significantly improve both performance and interpretability.

S.7 Supplementary Methods: Model costs

The total costs of these experiments are divided into three categories. Creating a fine-tuning dataset using reverse-chain-of-thought (RECoT) prompting, fine-tuning itself, and inference. In the RECoT step, we have employed the SOTA foundation models at the time, like GPT-4o and Claude Sonnet-3.5, and to generate a dataset of approximately 1700 chain-of-thought responses, it cost approximately $25 for GPT-4o, and $30 for Sonnet-3.5. The fine-tuning process cost approximately $1.6 for each model with 1epoch and a learning rate of 1.8. These are the recommended settings by OpenAI when the files were uploaded to their fine-tuning dashboard. The cost of inference for Claude Sonnet models is approximately

Sno	Model	Inference Cost	Breakdown
1	GPT-4o	$26.42	Uncached input: $1.52<br>Cached input: $13.00<br>Output: $11.90
2	Claude Sonnet 3.5	$17.69	Input: $0.79<br>Prompt caching write (5m): $0.01<br>Prompt caching read: $3.18<br>Output: $13.71
3	CARDS-mini-GPT-2024-12-05	$2.92	Uncached input: $0.19<br>Cached input: $1.44<br>Output: $1.29

Continued on next page

Table S4 – continued from previous page

Sno	Model	Inference Cost	Breakdown
4	CARDS-mini-GPT	$2.90	Uncached input: $0.18<br>Cached input: $1.44<br>Output: $1.27
5	CARDS-mini-Sonnet	$2.56	Uncached input: $0.18<br>Cached input: $1.44<br>Output: $0.93
6	CARDS-mini-Sonnet-2024-12-05	$2.48	Uncached input: $0.18<br>Cached input: $1.45<br>Output: $0.85
7	GPT-4o-Mini	$1.41	Uncached input: $0.09<br>Cached input: $0.72<br>Output: $0.59

Supplementary References

[1] Julia Bingler et al. *How Cheap Talk in Climate Disclosures Relates to Climate Initiatives, Corporate Emissions, and Reputation Risk*. Working paper. Available at SSRN 3998435, 2023.

[2] Yuntian Deng et al. *Implicit Chain of Thought Reasoning via Knowledge Distillation*. 2023. arXiv: 2311.01460 [cs.CL]. URL: https://arxiv.org/abs/2311.01460.

[3] Geoffrey Hinton, Oriol Vinyals, and Jeff Dean. *Distilling the Knowledge in a Neural Network*. 2015. arXiv: 1503.02531 [stat.ML]. URL: https://arxiv.org/abs/1503.02531.

[4] Jeffrey B Lewis et al. "Digital boundary definitions of united states congressional districts, 1789-2012". In: *Data file and code book* (2013).

[5] Liunian Harold Li et al. "Symbolic Chain-of-Thought Distillation: Small Models Can Also "Think" Step-by-Step". In: *Proceedings of the 61st Annual Meeting of the Association for Computational Linguistics (Volume 1: Long Papers)*. Ed. by Anna Rogers, Jordan Boyd-Graber, and Naoaki Okazaki. Toronto, Canada: Association for Computational Linguistics, July 2023, pp. 2665–2679. DOI: 10.18653/v1/2023.acl-long.150. URL: https://aclanthology.org/2023.acl-long.150/.
